# Whole brain MR spectroscopy in patients with anti-NMDAR encephalitis

**DOI:** 10.3389/fneur.2026.1892827

**Published:** 2026-09-24

**Authors:** Dominica Hudasch, Nima Mahmoudi, Xenyia Lisovska, Kaweh Pars, Tim Wollenweber, Patrick Nösel, Heinrich Lanfermann, Kurt-Wolfram Sühs, Xiao-Qi Ding

**Affiliations:** 1 Department of Neurology, Hannover Medical School, Hannover, Germany; 2 Institute of Diagnostic and Interventional Neuroradiology, Hannover Medical School, Hannover, Germany; 3 Department of Neuroradiology, Charité - Universitätsmedizin Berlin, Corporate Member of Freie Universität Berlin, Humboldt-Universität zu Berlin, Berlin, Germany; 4 Clinic for Anesthesiology and Intensive Care, Hannover Medical School, Hannover, Germany; 5 Department of Neurology, European Medical School, University Oldenburg, Oldenburg, Germany; 6 BG Klinikum Unfallkrankenhaus Berlin, Berlin, Germany

**Keywords:** anti-NMDAR encephalitis, autoimmune encephalitis, CNS inflammation, neuroinflammation, wbMRSI

## Abstract

**Objective:**

To investigate neurometabolic alterations in patients who have had anti-*N*-methyl-D-aspartate-receptor (NMDAR) encephalitis using whole-brain magnetic resonance spectroscopic imaging (wbMRSI) in comparison to matched healthy volunteers with no history of anti-NMDAR encephalitis.

**Methods:**

Twenty patients with a history of anti-NMDAR encephalitis and 20 healthy controls underwent wbMRSI. The concentrations of the metabolites N-acetyl-aspartate (NAA), choline (Cho), total creatine (tCr), Myo-Inositol (ml) and Glutamate/Glutamine (Glx) were measured in both frontal lobes (LFL and RFL), both temporal lobes (LTL and RTL), both parietal lobes (LPL and RPL), both occipital lobes (LOL and ROL), and cerebellum and compared using two-sided *t*-tests. Additionally, each patient underwent examinations including cognitive tests (DemTect), life quality assessment (SF36) and depressive disorders (BDI II) as fine motor skills (HPT) and walking speed (timed 25-foot walk test) investigations.

**Results:**

Exploratory analyses, uncorrected for multiple comparisons, revealed region specific metabolic differences including reduced NAA concentrations in the RTL, as well as an increase of Cho in the LFL, LPL, ROL and LOL. Concurrently, increased tCr was observed in the LFL and LOL, and elevated mI was detected in the LPL, ROL and LOL. Choline concentration in the ROL correlated with the left HPT and the 25-foot walk test. Conversely, the myo-inositol concentration in the ROL correlated with the NEOS score. Anti-NMDAR encephalitis patients showed higher depression values and lower DemTect results.

**Conclusion:**

Our findings indicate alterations in metabolite concentrations following anti-NMDAR encephalitis, as well as cognitive deficits and depressive symptoms. The differences in metabolite concentrations were not confined to the limbic system, and may therefore reflex more widespread processes that persist even after the initial symptoms have subsided. Given the uncorrected statical analyses, these findings should be considered hypothesis-generating and require confirmation in larger independent cohorts.

## Introduction

Anti-NMDAR encephalitis is the most common form of antibody-mediated encephalitis. Prodromal symptoms reminiscent of a viral infection may occur over a few days. The majority of patients develop psychiatric disturbances such as personality and behavioral changes, aggressive outbursts, paranoia or hallucinations. Within days to weeks, epileptic seizures, altered consciousness or central hypoventilation may occur. These conditions often require intensive medical care (1, 2). Treatment consists of high-dose steroids, plasma exchange and/or intravenous immunoglobulins and maintenance treatment with rituximab or cyclophosphamide. In most cases, patients regain sufficient functionality to carry out daily tasks independently (1).

However, a part of the patients suffers from persistent cognitive deficits after recovery from acute symptoms of anti-NMDAR encephalitis, which include not only memory, but also attention or executive function (3). For example, a longitudinal study of 40 patients with proven anti-NMDAR encephalitis showed that after recovering from the acute symptoms within an average of 2.3 years all patients had cognitive deficits, and half of the patients had severe cognitive impairment (4). This causes enormous suffering in the patient population after the acute phase has passed, even though the symptoms of encephalitis have subsided. The pathophysiological mechanism underlying the persistence of symptoms or deficits remains to be elucidated. Conventional cranial MRI imaging rarely yields correlating findings. In the acute phase of anti-NMDAR encephalitis, MRI abnormalities in the brain are detected in less than half of all cases, and in most cases mesiotemporally. These abnormalities subside after the acute phase, therefore conventional brain MR imaging appears to be only useful during this phase (5). A study combining T1- and T2-weighted MRI scans with resting-state functional MRI scans showed that functional connectivity in the hippocampus was reduced bilaterally, which was consistent with the patients’ memory performance. Furthermore, the white matter changes detected by diffusion tensor imaging (DTI) have been shown to correlate with the severity of the disease (2).

Supplementary spectroscopy provides information about metabolic processes in the brain regions under investigation and can reveal biochemical changes that are not visible on conventional imaging of the brain substance. Magnetic resonance spectroscopy can be used to determine concentrations of brain metabolites. This information can be used to assess neuronal integrity (N-Acetylaspertat/NAA), cellular membrane turnover (total choline/tCh), energy metabolism (total creatine/tCr), excitatory neurotransmitter/amino acid levels (glutamate and glutamine/Glx) or gliosis (myo-inositol/mI) (6). On this basis, statements can be made about malignancy of tumors or an assessment of damage or intactness of the brain tissue following ischemic, infectious or autoimmune processes (7–9).

A recently established whole-brain magnetic resonance spectroscopic imaging (wbMRSI) technique provides the possibility to simultaneously measure brain metabolites of the whole brain *in vivo* in one scan (10, 11).

Other studies already displayed the advantages of this technique in analyzing brain structures at the same time in neurological diseases, as outlined below.

In wbMRSI-studies of patients with Parkinson’s disease revealed changes in certain brain metabolites that affected all brain lobes. In addition, a lateral dominance of the changes was found, contralateral to the affected side of clinical expression (12). These findings demonstrated the feasibility of the wbMRSI method in revealing additional pathological changes in brain metabolism that are invisible with conventional MR imaging methods.

For example, conventional MRI imaging could reveal unspecific regional brain atrophy visible that may indicate possible cognitive deficits, but systematic analysis of brain metabolites in anti-NMDA-R encephalitis patients is not yet available.

Given the central role of NMDA receptors in glutamatergic neurotransmission, it is conceivable that anti-NMDAR encephalitis is associated with alterations in cerebral glutamatergic metabolism, which could potentially be identified using MRS-derived Glx levels. However, beyond glutamatergic dysfunction, the disease involves complex neuronal and inflammatory processes that may affect other metabolites too. NAA may reflect the neuronal dysfunction associated with the disease. Meanwhile mI may provide information relating to glial and inflammatory processes. Changes in Cho may indicate alterations in membrane turnover, whereas tCr relates to cerebral energy metabolism and is commonly used as a metabolic reference (6). As anti-NMDAR encephalitis affects distributed neuronal networks rather than a single, anatomically confined brain region, wbMRSI could identify spatially heterogeneous metabolic alterations missed by conventional single-voxel spectroscopy (13).

In the case of obvious cognitive deficits and evidence of depressive symptoms, despite absent or mild clinical neurological deficits without interference with activities of daily living, it seems important to more deeply assess the underlying structural changes of the central nervous system. The present study therefore aimed to investigate potential alterations in NAA, Cho, tCr, mI and Glx across multiple brain regions in patients following anti-NMDAR encephalitis, compared with healthy controls.

## Materials and methods

### Patient data and clinical investigation

The Ethics Committee of the Hannover Medical School approved the human studies (No. 6167–2019). Written informed consent was obtained from all subjects.

The diagnosis of anti-NMDAR encephalitis was made according to the Graus criteria on the basis of symptoms, positive antibody detection at least in the cerebrospinal fluid, and with the aid of EEG and MRI (14). The subjects included in the study are 20 patients with anti-NMDAR encephalitis and 20 age and sex-matched healthy volunteers as controls. Patients who participated in our study were treated as inpatients at the Department of Neurology, Hannover Medical School, the Department of Neurology, University Hospital Oldenburg and/or in the neuroimmunological outpatient clinic of the Hannover Medical School. Patients aged 18 years and older, with a confirmed diagnosis of anti-NMDAR encephalitis and antibody detection at least in the CSF, were included. Concomitant diseases included bronchial asthma (*n* = 1), Hashimoto’s thyroiditis (*n* = 1), cholecystolithiasis (*n* = 1) and Sjögren’s syndrome (*n* = 1). Patients currently receiving neuroleptic treatments were excluded.

Each patient completed a Beck’s Depression Inventory (BDI) and a Short Form 36 (SF36) questionnaire. In addition, a neurologist assessed a DemTect to quantify cognitive impairment. A nine-hole peg test (9hpt) and a 25-foot (7.62 m) walk test were performed, too.

### MR examinations and MR-analyses

All patients received a MR examination using a 12-channel phased-array head coil at 3 T (Verio, Siemens, Erlangen, Germany), all sequences were aligned at the same angle. As described before, the MRI protocol contained clinical relevant sequences such as T1- and T2w- sequences, a T2w-FLAIR sequence for morphological evaluation, an isotropic (1 × 1 × 1 mm^3^) 3D-T1 MPRAGE (magnetization-prepared rapid gradient echo), and a volumetric spin-echo planar spectroscopic imaging (EPSI) sequence (relaxation time (TR)/echo time (TE) = 1550/17.6 ms, 50 × 50 voxels in-plane and 18 slices, over a field of view of 280 × 280 × 180 mm^3^, acquisition time: about 17 min) for wbMRSI (15). Manual shimming was performed prior to the EPSI acquisition. Additionally, a second EPSI sequences was acquired to obtain MRSI data without water suppression, which was then used for processing functions and providing internal reference for the normalization of the metabolic data, measurement and correction of the resonance frequency offset at each voxel location and correction of line shape distortions. Only CSF partial-volume correction was applied; no separate correction for grey/white matter composition was performed, given the small magnitude of the observed nominal tissue-composition differences between groups (Table 1) and for consistency with previous applications of this technique (11, 12, 15, 16).

**Table 1:** Tissue compositions and linewidth in individual brain regions were measured by whole-brain MR spectroscopy.

	LW						CSF				
Brain region	*n*	Patients		Controls		*p*	Patients		Controls		*p*
		Mean	SD	Mean	SD		Mean	SD	Mean	SD	
RFL	20	7.35	0.45	7.29	0.58	0.69	0.08	0.01	0.07	0.01	0.18
LFL	20	7.32	0.55	7.15	0.47	0.31	0.08	0.01	0.08	0.01	0.42
RTL	20	7.66	0.39	7.61	0.53	0.74	0.06	0.02	0.05	0.02	0.10
LTL	20	7.55	0.56	7.26	0.31	**0.05**	0.07	0.02	0.06	0.02	0.34
RPL	20	6.91	0.78	6.53	0.61	0.09	0.08	0.02	0.08	0.02	0.62
LPL	20	6.85	0.87	6.54	0.58	0.19	0.09	0.01	0.08	0.02	0.71
ROL	20	6.57	0.50	6.46	0.56	0.50	0.06	0.02	0.06	0.02	0.63
LOL	20	6.58	0.50	6.38	0.57	0.25	0.06	0.02	0.06	0.03	0.78
Cbl	20	7.45	0.69	7.51	0.72	0.79	0.07	0.01	0.07	0.01	0.56

		GM					WM				
Brain region	*n*	Patients		Controls		*p*	Patients		Controls		*p*
		Mean	SD	Mean	SD		Mean	SD	Mean	SD	
RFL	20	0.41	0.04	0.43	0.02	**0.05**	0.50	0.04	0.48	0.02	0.06
LFL	20	0.41	0.04	0.44	0.02	**0.02**	0.50	0.03	0.48	0.02	**0.01**
RTL	20	0.53	0.03	0.55	0.03	0.20	0.3	0.03	0.38	0.03	0.46
LTL	20	0.52	0.03	0.54	0.02	0.05	0.39	0.02	0.38	0.02	0.09
RPL	20	0.43	0.04	0.45	0.03	0.07	0.48	0.03	0.45	0.03	0.03
LPL	20	0.40	0.04	0.43	0.02	0.05	0.50	0.04	0.48	0.02	**0.03**
ROL	20	0.54	0.04	0.55	0.03	0.35	0.38	0.03	0.36	0.03	0.25
LOL	20	0.53	0.04	0.54	0.02	0.62	0.39	0.03	0.38	0.03	0.58
Cbl	20	0.68	0.04	0.68	0.04	0.99	0.24	0.04	0.24	0.04	0.75

Mean values were compared between the patient group and the control group.

Paired *t*-test was used for the analysis (*p < 0.05, **p < 0.01, ***p < 0.001, ****p = 0.000). Statistical significance is indicated by bold font. Reported *p*-values are uncorrected for multiple comparisons.

Cerebellum (CBL), Cerebrospinal Fluid (CSF), Grey Matter (GM), Left Frontal Lobe (LFL), Left Occipital Lobe (LOL), Left Parietal Lobe (LPL), Left Temporal Lobe (LTL), Light Width (LW), Right Frontal Lobe (RFL), Right Occipital Lobe (ROL), Right Parietal Lobe (RPL), Right Temporal Lobe (RTL), Standard Deviation (SD), White Matter (WM).

As stated in previous publication, the EPSI data, along with the MPRAGE data, were investigated using the software package Metabolic Imaging and Data Analysis System (MIDAS) to obtain volumetric metabolite maps (10). The processing included zero-filling to 64 × 64 × 32 points and spatial smoothing, resulting in an interpolated basic voxel volume of 0.107 mL (4.375 × 4.375 × 5.625 mm^3^) and an effective voxel volume of 1.5 mL (12). This analysis was conducted to create brain maps of the metabolites NAA, Cho, tCr, Glx, mI, and the corresponding spectral linewidth (LW). Moreover, the analysis was used to generate maps of the relative cerebrospinal fluid (CSF) component, grey matter, and white matter.

All the maps were thereafter subjected to spatial transformation and interpolation to provide a uniform spatial reference with a 2-mm isotropic resolution. Metabolite concentrations, including NAA, Cho, tCr, Glx and mI, were calculated as the ratio of the metabolite signal to the internal, non-water-suppressed water reference signal acquired with identical TR/TE, without correction for T1 or T2 relaxation effects of water or metabolite signals. Reported values therefore represent relative institutional units (i.u.) rather than absolute molar concentrations, consistent with previous applications of this technique (11, 12, 15, 16). The processing also included calculation of the fractional tissue volume contributing to each MRSI voxel, which used a tissue segmentation. This reference was linked to an atlas that delineated the several brain lobes and the cerebellum. Measurements were obtained using multivoxel analysis based on nine atlas-defined anatomical regions: frontal lobe left (LFL) and frontal lobe right (RFL), temporal lobe left (LTL) and temporal lobe right (RTL), parietal lobe left (LPL) and parietal lobe right (RPL), occipital lobe left (LOL) and occipital lobe right (ROL); and the cerebellum and brainstem (Cbl); which was done in similar fashion as previous research projects (12, 16). In order to account for the partial volume of CSF, a correction was made for the contribution of CSF volume according the formula *Met’ = Met/(1–f_csf_)*, where *Met* represents the uncorrected metabolite value and *f_csf_* represents the fractional volume of CSF (FVCSF) in the MRSI voxel. It should be noted that this correction is applicable for values of *f_csf_* between 0 and 0.3; Voxels with *f_csf_* **>** 0.3 or LW > 12 Hz were not included in the computation (10). The MRSI data of 20 healthy persons, who were based on sex, age and BMI and this one-to-one pairing was retained in all paired statistical comparisons. These subjects were included in prior research and their data was gathered using the same MR collection and imaging technique (11, 15).

The T1w, T2w, and FLAIR images were inspected by neuroradiologists to screen incidental findings.

### Statistical analysis

A comparison was made between patients after anti-NMDAR encephalitis and 20 healthy control subjects. A Levene’s test was conducted to assess the equality of variables. Equal variances were found for age and BMI. Fisher’s exact test was used to examine the comparability of sex distribution between patients and healthy controls. In more than 90% of the comparisons of the concentrations of the five metabolites analyzed, there was homogeneity of variance (*p* > 0.05).

This did not apply to the comparisons of the DemTect and BDI II results. A paired *t*-test was used to compare the measured lobar metabolites of the patients and the control. A nonparametric Spearman’s correlation test was used to estimate possible correlations between the concentrations of lobar metabolites and clinical tests, including the nine-hole peg test, timed 25-foot walk test, short-form 36 health survey (SF36), or anti-NMDAR Encephalitis One-Year Functional Status (NEOS) score.

Statistical significance was defined as a two-sided *p*-value < 0.05. Due to the exploratory character of the study and the relatively small sample size, correction for multiple comparisons was not applied in order to avoid an overly conservative reduction in statistical power. Accordingly, the results should be interpreted as exploratory and hypothesis-generating.

Statistical analyses were performed with SPSS version 26 (SPSS IBM, New York, USA).

## Results

### Patient characteristics

Demographic aspects, an overview of symptoms, laboratory parameters and important data for further diagnosis, as well as the results of clinical tests performed with the patient groups in addition to wbMRSI, are summarized in Table 2. The control group for comparison was matched for sex (female *n* = 15; 75%), age (*n* = 20; mean 32 years, range 20–63 years), and BMI (*n* = 20; mean 24.41, range 16.98–37.04).

**Table 2:** The demographic aspects, clinical findings, instrumental findings and cerebrospinal fluid chemistry findings are presented.

	*n*/%	Mean	SD	Min	Max
Female *n*; %	15; 75%				
Age at diagnosis, years	*n* = 18	31.6	13.53	18.4	63.7
Age at scan, years	*n* = 20	32.6	13.17	20	63.7
BMI	*n* = 20	24.41	4.1	16.98	37.04
Time between diagnosis and scan, months	*n* = 18	28.6	20.7	0.5	72
Psychiatric symptoms	76.5% (13; *n* = 17)				
Seizures	59% (10; *n* = 17)				
ICU treatment in the acute phase of disease	24% (4; *n* = 17)				
mRS at peak of disease	*n* = 17	3.5	1.07	1	5
mRS at scan	*n* = 17	1.2	0.95	0	3
EEG: uni/bilateral temporal slowing	37.5% (6; *n* = 16)				
Abnormalities in the MRI scan indicative for encephalitis	18% (3; *n* = 17)				
Elevated cell count at diagnosis (≥5 cells/μL)	59% (10; *n* = 17)				
Mean CSF cell count at diagnosis (cells/μl)	*n* = 17	23.4	42.72	1	150
Mean CSF protein at diagnosis (mg/L)	*n* = 17	350	110.9	180	552
Positive OCB at diagnosis	64.7% (11; *n* = 17)				
BDI II	*n* = 19	8.6	7.259	0	26
DemTect	*n* = 19	14.3	4.254	4	18
SF36 PF	*n* = 19	89.21	17.58	40	100
SF36 RP	*n* = 19	65.79	40.15	0	100
SF36 RE	*n* = 19	66.67	43.03	0	100
SF36 E	*n* = 19	53.68	20.6	20	90
SF36 WB	*n* = 19	70.95	19.39	32	96
SF36 SF	*n* = 19	74.47	28.35	0	100
SF36 P	*n* = 19	86.71	21.86	32.5	100
SF36 GH	*n* = 19	62.11	21.66	15	100
SF36 HC	*n* = 19	56.58	31	0	100
Left nine-hole peg test	*n* = 19	17.86	5.408	1,243	30.1
Right nine-hole peg test	*n* = 19	16.83	5.473	9.57	26.7
Timed 25-foot-walk test	*n* = 19	6.7	1.865	4.04	9.6
NEOS	*n* = 18	2	0.9701	0	4

In addition, an overview of the results of the scorings carried out on the patients included is provided. Anti-NMDA Encephalitis One-Year-Outcome-Score (NEOS-Score), Becks Depression Inventory (BDI), bodily pain (SF-36-P), body mass index (BMI), cerebrospinal fluid (CSF), dementia detection test (DemTect), electroencephalography (EEG), general health (SF-36-GH), health change (SF-36-HC), intensive care unit (ICU), magnetic resonance imaging (MRI), mental health (well-being) (SF-36-WB), Modified Rankin Scale (mRS), oligoclonal bands (OCB), physical functioning (SF-36-PF), role limitations due to emotional problems (SF-36-RE), role limitations due to physical health (SF-36-RP), social functioning (SF-36-SF), standard deviation (SD), Timed 25-Foot Walk Test (TWT), vitality (energy/fatigue) (SF-36-E).

In the anti-NMDAR encephalitis group psychiatric symptoms were observed in over three-quarters of the patients, while slightly more than half of the patients exhibited epileptic seizures. Intensive care was administered to approximately a quarter of all patients in the anti-NMDAR encephalitis group. Notably, less than 20% of the patients demonstrated encephalitis-typical changes in conventional brain MRI imaging. The average BDI II score was found to be slightly below 9 points, which corresponds to a mild depressive disorder. A closer look at the BDI II scores showed that two of the anti-NMDAR encephalitis patients had minimal depressive symptoms (9 and 10 points), three patients had mild depression scores (14–19 points) and two patients were moderately depressed, according to the score (20 and 26 points). For the remaining patients, no significant depressive symptoms could be inferred from the score. The mean score on the DemTect was 14, which corresponds to the lower limit of a normal result. In five patients, the DemTect indicated cognitive impairment (4–12 points).

Compared with the control group, the anti-NMDAR encephalitis patients scored significantly higher (*p* = 0.001) on the depression test and significantly lower on the DemTect (*p* = 0.001) (Figure 1A). The exemplary motor examination using the 25-foot walk test yielded a median walking time of 6.2 s. In a large, healthy reference cohort, the median time taken to complete the test was 3.7 s, with times ranging from 2.8 to 5.2 s (17). The median time recorded for the right 9-hole peg test was 14.14 s, which is 2.51 s below the stated median value for the right hand in the 25–29 years age group. Meanwhile, the median time recorded for the left 9-hole peg test was 15.37 s, which is 2.33 s below the quoted median value for the left side for the 25–29 year age group (18). These differences were small and are reported for descriptive purposes only. No statistical comparison was performed with the normative cohort.

**Figure 1:**
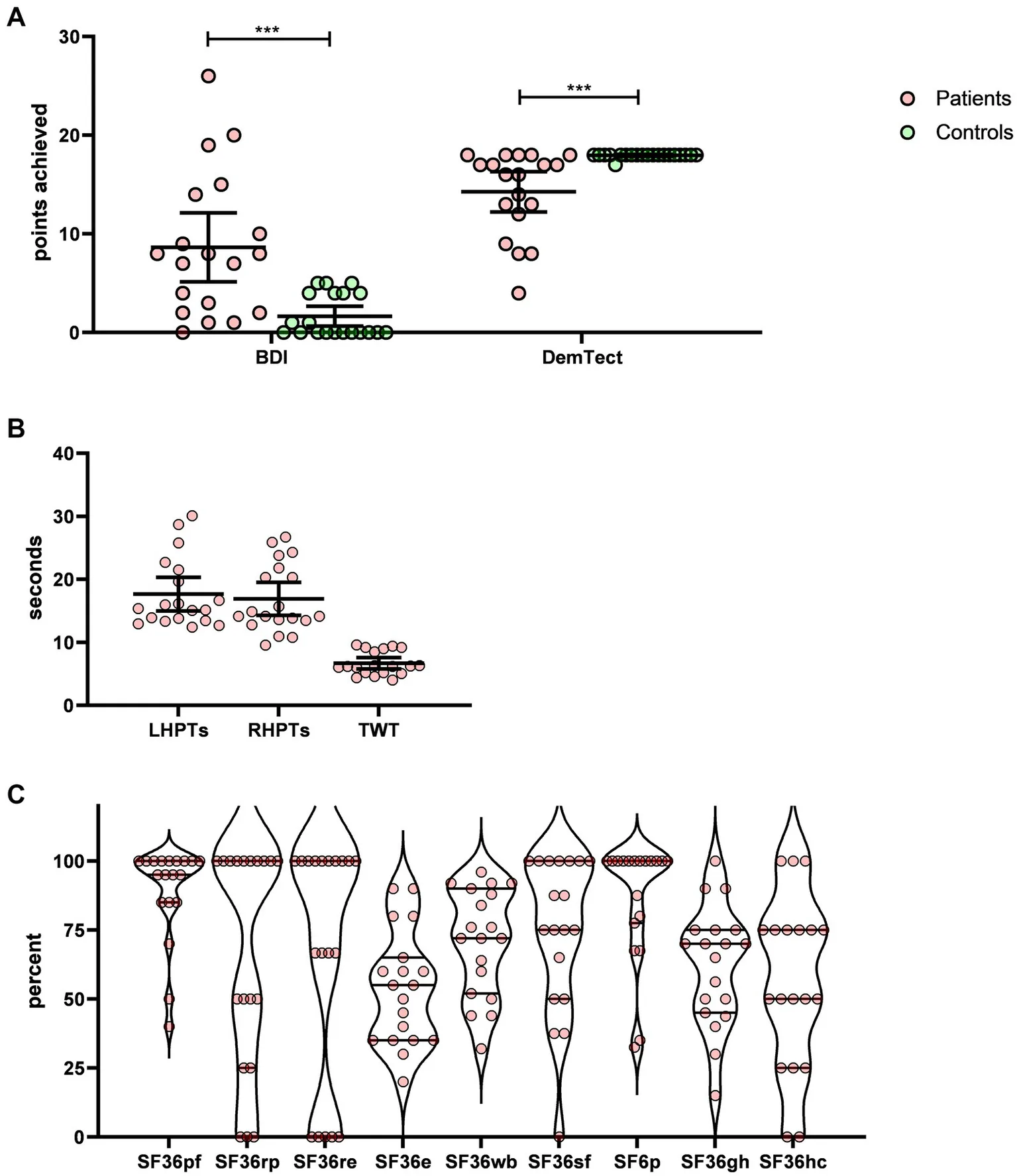
A comparison of the scores achieved by the patient group and the control group on the BDI II and DemTect is presented herewith **(A)**. Presentation of the durations of the 9-hole peg tests per side and the TWT **(B)** as well as the presentation of the results of the SF36 broken down by individual aspects **(C)** for the patient group. Paired *t*-test was used for the performed analyses (*p < 0.05, **p < 0.01, ***p < 0.001, ****p = 0.000). Becks Depression Inventory (BDI), Bodily Pain (SF-36-P), Dementia Detection Test (DemTect), General Health (SF-36-GH), Health Change (SF-36-HC), Left-Hand 9-Hole-Peg-Test (L-HPT), Mental Health (Well-being) (SF-36-WB), Physical Functioning (SF-36-PF), Right-Hand 9-Hole-Peg-Test (R-HPT), Role Limitations due to Emotional Problems (SF-36-RE), Role Limitations due to Physical Health (SF-36-RP), Social Functioning (SF-36-SF), Timed 25-Foot Walk Test (TWT), Vitality (Energy/Fatigue) (SF-36-E).

No significant differences were observed between the right- and left-hand performance within the patient group (*p* = 0.673) (Figure 1B). The results of the subjective assessment of quality of life (SF36) demonstrate a relatively wide range of responses across the various domains (Figure 1C). Overall, patients demonstrated high levels of physical functioning and low pain impairment (mean PF: 89.21; P: 86.71). Energy level (mean E: 53.68) and general health perception (mean GH: 62.11) were in the medium range. Emotional and social functioning (mean RE: 66.67; mean SF: 74.47) and psychological well-being (mean WB: 70.95) were slightly below those of healthy comparison groups (19). Perceived change in health (mean HC: 56.58) indicates moderate improvement or stability compared to the previous year.

### Brain metabolite concentrations

The following section presents the concentrations of regional metabolites measured in nine brain regions in patients following anti-NMDAR encephalitis and in healthy volunteers. These results are displayed in Table 3, alongside the results of the *t*-test analysis of metabolite distributions. Example metabolite maps of NAA, Cho, tCr, mI, and Glx of a 24 years old male volunteer derived from wbMRSI data is shown in Figure 2. Cho demonstrated the most prevalent disparities between the two groups, exhibiting significantly elevated values in four brain regions of the anti-NMDAR encephalitis group in comparison to the control group. The increase was observed in the left hemisphere (frontal, parietal and occipital) as well as in the right occipital lobe. Concurrently, the average mI level was found to be increased in three lobes of the anti-NMDAR encephalitis group (left parietal and occipital on both sides). Furthermore, a differential analysis of the tCr content revealed variations in two lobes, with increased concentrations observed in the left frontal and left occipital regions in patients following anti-NMDAR encephalitis. Conversely, the average NAA concentration was lower in one lobe of the brain (right temporal lobe) in the anti-NMDAR encephalitis group compared to the control group. No significant difference in Glx concentration was observed between the patient and control groups.

**Table 3:** The concentration of metabolites, as measured by whole-brain MR spectroscopy, is represented in the individual brain regions.

Brain region	NAA						Cho					ml				
	*n*	Patients mean ± SD	Controls mean ± SD	Mean difference (95% CI)	Cohen’s *d* (95% CI)	*p*	Patients mean ± SD	Controls mean ± SD	Mean difference (95% CI)	Cohen’s *d* (95% CI)	*p*	Patients mean ± SD	Controls mean ± SD	Mean difference (95% CI)	Cohen’s *d* (95% CI)	*p*
RFL	20	12.11 ± 0.87	12.41 ± 0.59	−0.30 (−0.78 to 0.17)	−0.41(−1.033 to 0.22)	0.204	2.33 ± 0.22	2.22 ± 0.25	0.11(−0.04 to 0.26)	0.46(−0.17 to 1.09)	0.152	4.05 ± 0.63	3.89 ± 0.57	0.15(−0.23 to 0.54)	0.26(−0.37 to 0.88)	0.423
LFL	20	12.31 ± 0.77	12.5 ± 0.67	−0.2(−0.66 to 0.26)	−0.27(−0.9 to 0.35)	0.392	2.45 ± 0.28	2.23 ± 0.21	0.22(0.07 to 0.38)	0.91(0.25 to 1.56)	**0.007**	4.45 ± 0.76	4.11 ± 0.63	0.34(−0.11 to 0.78)	0.48(−0.15 to 1.11)	0.135
RTL	20	10.01 ± 0.94	10.62 ± 0.55	−0.60(−1.10 to −0.11)	−0.78(−1.42 to −0.14)	**0.019**	1.95 ± 0.13	1.96 ± 0.21	−0.01(−0.12 to 0.11)	−0.03(−0.65 to 0.59)	0.92	3.93 ± 0.46	3.73 ± 0.36	0.20(−0.06 to 0.47)	0.49(−0.15 to 1.11)	0.133
LTL	20	10.87 ± 0.78	11.14 ± 0.65	−0.27(−0.73 to 0.19)	−0.37(−0.99 to 0.26)	0.248	2.16 ± 0.213	2.06 ± 0.17	0.10(−0.03 to 0.22)	0.50(−0.14 to 1.12)	0.126	4.58 ± 0.75	4.2 ± 0.36	0.37(−0.003 to 0.75)	0.64(−0.01 to 1.27)	0.055
RPL	20	12.25 ± 0.79	12.53 ± 0.53	−0.27(−0.70 to 0.16)	−0.40(−1.03 to 0.23)	0.21	1.94 ± 0.2	1.83 ± 0.2	0.11(−0.02 to 0.24)	0.54(−0.09 to 1.17)	0.094	3.59 ± 0.54	3.43 ± 0.54	0.15(−0.19 to 0.50)	0.28(−0.34 to 0.90)	0.376
LPL	20	12.57 ± 0.82	12.64 ± 0.69	−0.07(−0.56 to 0.41)	−0.10(−0.72 to 0.52)	0.76	2.04 ± 0.19	1.89 ± 0.22	0.15(0.02 to 0.28)	0.73(0.09 to 1.37)	**0.026**	3.9 ± 0.63	3.41 ± 0.41	0.48(0.15 to 0.82)	0.92(0.26 to 1.56)	**0.006**
ROL	20	12.04 ± 0.84	12.24 ± 0.62	−0.20(−0.68 to 0.27)	0.275(−0.90 to 0.35)	0.39	1.59 ± 0.14	1.47 ± 0.15	0.11(0.02 to 0.21)	0.76(0.11 to 1.39)	**0.022**	3.84 ± 0.47	3.54 ± 0.41	0.30(0.02 to 0.59)	0.69(0.04 to 1.32)	**0.036**
LOL	20	12.52 ± 0.72	12.49 ± 0.58	0.03(−0.39 to 0.45)	0.045(−0.58 to 0.66)	0.888	1.72 ± 0.19	1.57 ± 0.15	0.15(0.04 to 0.26)	0.85(0.19 to 1.49)	**0.011**	4.17 ± 0.54	3.7 ± 0.39	0.47(0.16 to 0.77)	0.999(0.34 to 1.65)	**0.003**
Cbl	20	10.51 ± 0.55	10.3 ± 0.86	0.21(−0.26 to 0.67)	0.29(−0.34 to 0.91)	0.372	2.83 ± 0.31	2.7 ± 0.26	0.13(−0.05–0.31)	0.45(−0.18 to 1.08)	0.161	4.65 ± 0.72	4.39 ± 0.49	0.25(−0.14 to 0.65)	0.41(−0.22 to 1.02)	0.199

Brain region	tCr						Glx				
	*n*	Patients mean ± SD	Controls mean ± SD	Mean difference (95% CI)	Cohen’s *d* (95% CI)	*p*	Patients mean ± SD	Controls mean ± SD	Mean difference (95% CI)	Cohen’s *d* (95% CI)	*p*
RFL	20	11.41 ± 0.56	11.1 ± 0.54	0.32 (−0.03 to 0.67)	0.58 (−0.06 to 1.21)	0.076	9.36 ± 0.55	9.24 ± 0.51	0.12 (−0.22 to 0.45)	0.22 (−0.40 to 0.84)	0.492
LFL	20	11.71 ± 0.72	11.11 ± 0.55	0.60 (0.19 to 1.01)	0.94 (0.28 to 1.60)	**0.005**	9.68 ± 0.66	9.66 ± 0.58	0.02 (−0.38 to 0.42)	0.03 (−0.59 to 0.65)	0.923
RTL	20	9.87 ± 0.53	9.92 ± 0.52	−0.04 (−0.38 to 0.29)	−0.08 (−0.70 to 0.54)	0.796	8.79 ± 0.75	8.85 ± 0.82	−0.07 (−0.57 to 0.43)	−0.09 (−0.71 to 0.53)	0.784
LTL	20	10.87 ± 0.73	10.5 ± 0.52	0.37 (−0.04 to 0.78)	0.58 (−0.05 to 1.21)	0.073	9.08 ± 0.64	9.08 ± 0.6	0.001 (−0.39 to 0.40)	0.002 (−0.62 to 0.62)	0.995
RPL	20	10.62 ± 0.68	10.56 ± 0.83	0.06 (−0.43 to 0.54)	0.08 (−0.55 to 0.70)	0.814	8.81 ± 0.88	9.24 ± 0.5	−0.43 (−0.89 to 0.03)	−0.60 (−1.23 to 0.04)	0.069
LPL	20	11.1 ± 0.7	10.63 ± 0.86	0.47 (−0.03 to 0.97)	0.60 (−0.04 to 1.23)	0.067	9.12 ± 0.8	9.41 ± 0.68	−0.30 (−0.77 to 0.18)	−0.40 (−1.02 to 0.23)	0.215
ROL	20	10.62 ± 0.59	10.44 ± 0.78	0.18 (−0.26 to 0.62)	0.26 (−0.36 to 0.88)	0.417	8.6 ± 0.7	8.79 ± 0.62	−0.19 (−0.62 to 0.23)	−0.29 (−0.91 to 0.33)	0.362
LOL	20	11.43 ± 0.86	10.75 ± 0.84	0.68 (0.14 to 1.23)	0.80 (0.15 to 1.44)	**0.016**	9.13 ± 0.74	8.97 ± 0.71	0.17 (−0.30 to 0.63)	0.23 (−0.39 to 0.85)	0.469
Cbl	20	14.34 ± 1.15	13.89 ± 1.38	0.45 (−0.36 to 1.26)	0.35 (−0.27 to 0.98)	0.27	9.68 ± 0.7	9.69 ± 0.73	−0.01 (−0.47 to 0.45)	−0.01 (−0.63 to 0.61)	0.965

The mean concentrations were compared between the patient group and the control group. Paired *t*-test was used for the analysis (*p < 0.05, **p < 0.01, ***p < 0.001, ****p = 0.000). Statistical significance is indicated by bold font. Reported *p*-values are uncorrected for multiple comparisons. Cerebellum (CBL), Cholin (Cho), Glutamate + Glutamine (Glx), Left Frontal Lobe (LFL), Left Occipital Lobe (LOL), Left Parietal Lobe (LPL), Left Temporal Lobe (LTL), Myo-Inositol (mI), Right Frontal Lobe (RFL), Right Occipital Lobe (ROL), Right Parietal Lobe (RPL), Right Temporal Lobe (RTL), Standard Deviation (SD), total Creatine (tCr).

**Figure 2:**
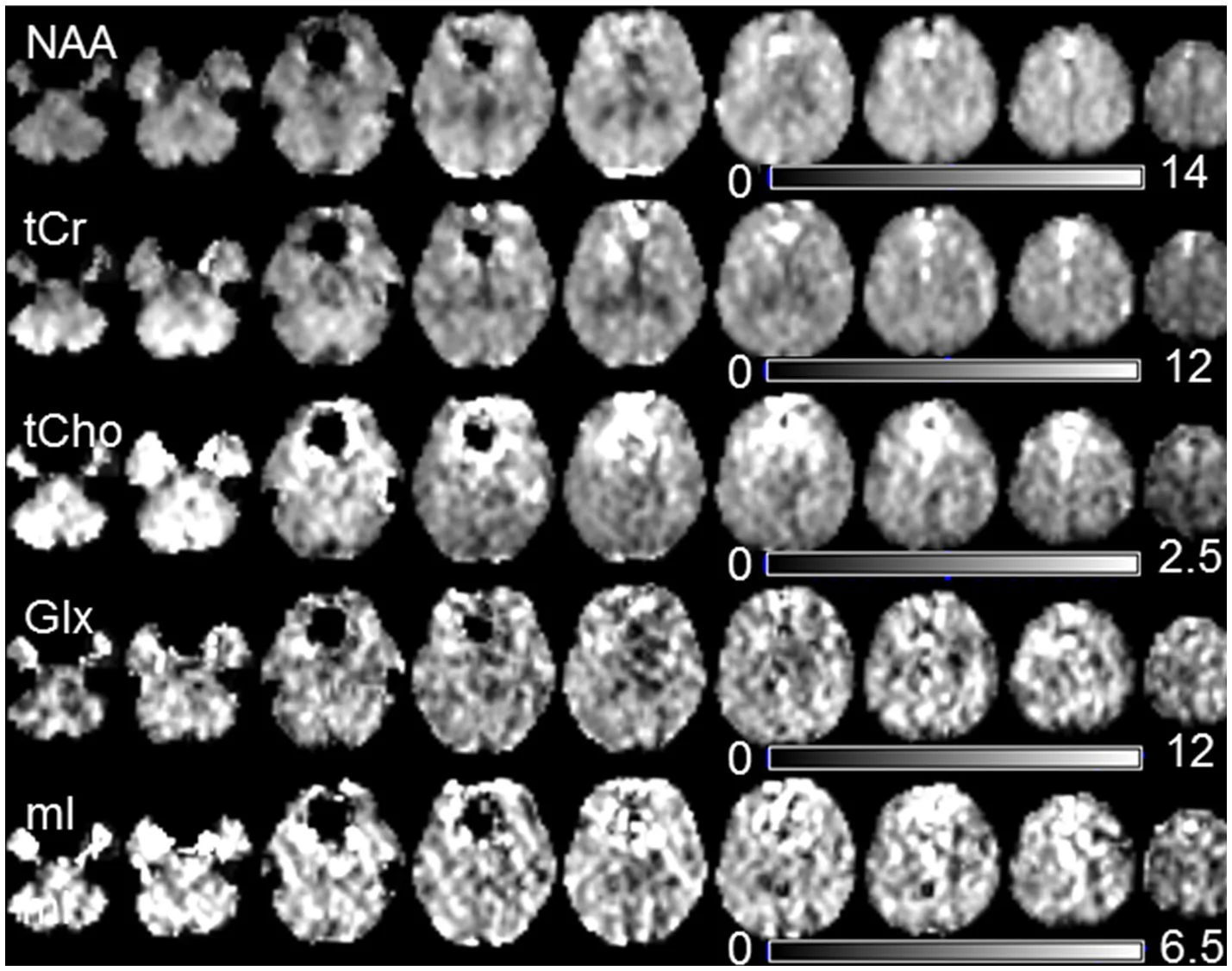
Example metabolite maps of NAA, Cho, tCr, mI, and Glx of a 24 years old male volunteer. Choline (Cho), Glutamate + Glutamine (Glx), Myo-Inositol (mI), N-Acetyl-Aspartate (NAA), total Creatine (tCr). The maps are shown for illustrative purposes to demonstrate MRSI data acquisition, processing, and spatial metabolite distribution and are not intended to represent group-level patient-control differences.

Figures 3A–E Illustrates the comparisons per brain region between anti-NMDAR encephalitis and healthy controls for each metabolite. A significantly broader spectral linewidth was observed in the left temporal lobe of patients in the anti-NMDAR encephalitis group, as opposed to those in the control group (Table 1).

**Figure 3:**
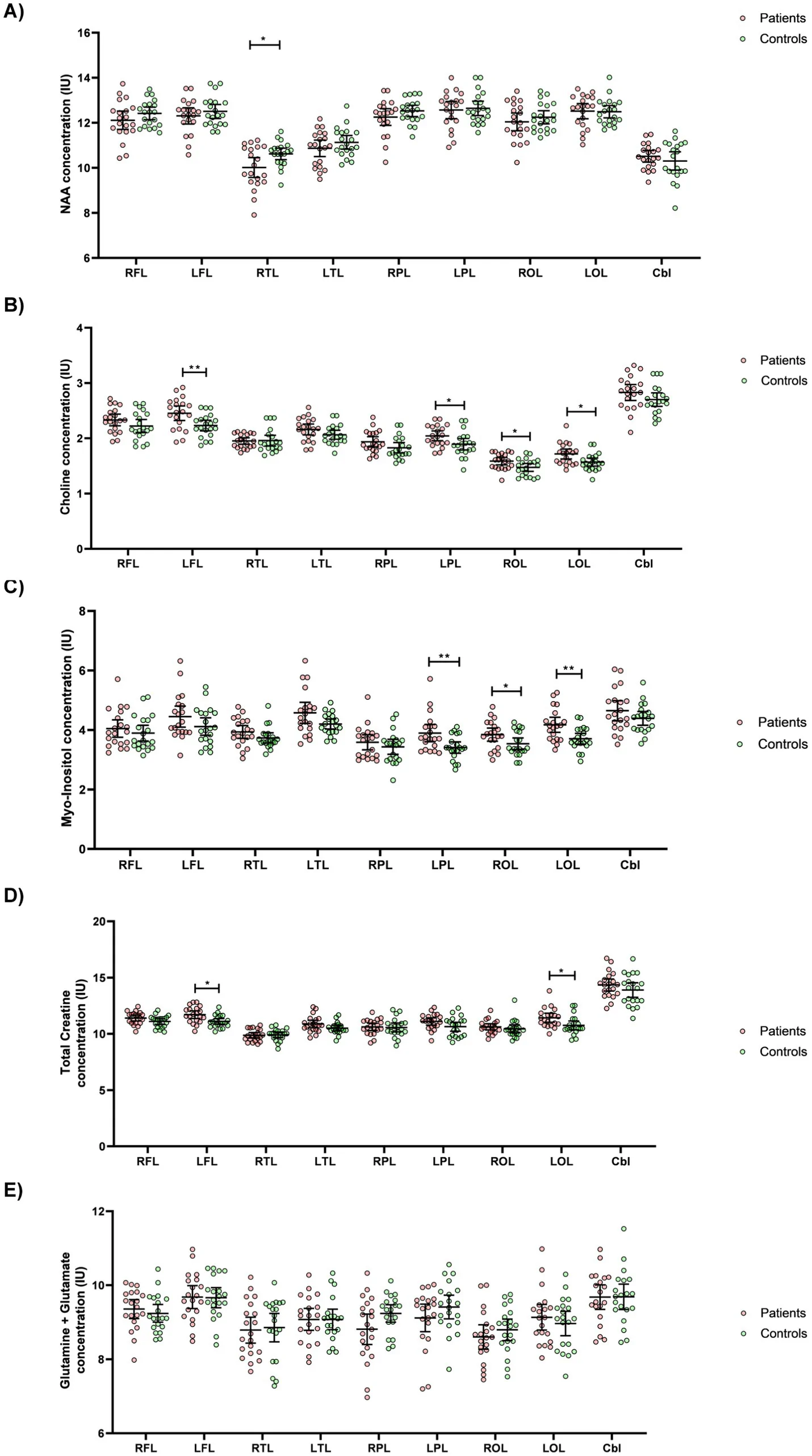
Concentrations of NAA **(A)**, Cho **(B)**, mIns **(C)**, tCr **(D)**, and Glx **(E)** for the respective brain regions measured in patients after anti-NMDAR encephalitis and healthy controls. Paired *t*-test was used for the analysis (*p < 0.05, **p < 0.01, ***p < 0.001, ****p = 0.000). Reported *p*-values are uncorrected for multiple comparisons. Cerebellum (CBL), Cholin (Cho), Glutamate + Glutamine (Glx), Left Frontal Lobe (LFL), Left Occipital Lobe (LOL), Left Parietal Lobe (LPL), Left Temporal Lobe (LTL), Myo Inositol (mI), N-cetyl-spartate (NAA), Right Frontal Lobe (RFL), Right Occipital Lobe (ROL), Right Parietal Lobe (RPL), Right Temporal Lobe (RTL), otal reatine (tCr).

The findings of the correlation analyses of the individual metabolites in the anti-NMDAR encephalitis group with nine-hole peg tests on both sides, a 25-foot walk test, the NEOS score and the individual items of the SF36 are demonstrated in Tables 4A, B and Supplementary Table S1A,B. It was demonstrated that there was a statistically significant correlation between the outcomes of the left nine-hole peg test and the Cho concentrations in the right occipital lobe (*R* = −0.47, *p* = 0.042). Moreover, the findings revealed a correlation between the walking speed on the 25-foot walk test and Cho concentration in the right occipital lobe (*R* = −0.554, *p* = 0.014). There was also a correlation between the concentration of ml in the right occipital lobe and the NEOS score in patients with anti-NMDAR encephalitis (*R* = 0.51, *p* = 0.031).

**Table 4:** A, B Presentation of the results of the nonparametric Spearman’s correlation test of the average concentration for NAA (A) and total Creatine (B) in the individual brain regions with the results of the clinical tests performed.

Clinical scores	NAA																		
	*n*	RFL		LFL		RTL		LTL		RPL		LPL		ROL		LOL		Cbl	
		*R*	*p*	*R*	*p*	*R*	*p*	*R*	*p*	*R*	*p*	*R*	*p*	*R*	*p*	*R*	*p*	*R*	*p*
A																			
L-HPT (s)	19	0.312	0.193	0.437	0.061	0.193	0.429	0.195	0.424	0.242	0.318	0.363	0.126	0.314	0.19	0.444	0.057	0.307	0.201
R-HPT (s)	19	0.248	0.305	0.353	0.138	0.181	0.459	0.221	0.363	0.176	0.472	0.435	0.062	0.302	0.209	0.405	0.086	0.106	0.665
TWT	19	0.158	0.518	0.327	0.171	0.094	0.702	−0.022	0.929	0.161	0.509	0.237	0.329	0.161	0.509	0.139	0.571	0.194	0.426
NEOS	18	0.024	0.926	−0.052	0.838	−0.266	0.286	−0.118	0.642	0.112	0.658	−0.127	0.329	−0.179	0.477	0.15	0.554	−0.291	0.242
SF36pf	19	0.313	0.192	0.2	0.412	0.414	0.078	0.303	0.208	0.135	0.58	0.256	0.29	0.307	0.201	0.327	0.172	**0.557**	**0.013**
SF36rp	19	0.173	0.479	0.016	0.947	0.278	0.249	0.119	0.626	−0.005	0.985	0.022	0.929	0.263	0.277	0.119	0.629	**0.569**	**0.011**
SF36re	19	0.2	0.411	0.005	0.984	0.219	0.368	0.008	0.975	−0.057	0.817	−0.009	0.972	0.218	0.371	0.112	0.649	**0.5**	**0.029**
SF36e	19	−0.114	0.642	−0.293	0.224	−0.226	0.352	−0.182	0.456	−0.24	0.322	−0.32	0.182	−0.186	0.447	−0.191	0.434	0.056	0.821
SF36wb	19	0.137	0.577	0.013	0.957	0.039	0.875	−0.045	0.855	−0.103	0.675	−0.194	0.427	0.025	0.92	−0.05	0.838	0.434	0.063
SF36sf	19	0.262	0.279	0.015	0.95	0.316	0.188	0.184	0.452	−0.024	0.921	0.016	0.947	0.347	0.146	0.11	0.653	**0.481**	**0.037**
SF6p	19	−0.155	0.526	−0.092	0.707	−0.098	0.689	−0.344	0.15	−0.103	0.673	−0.264	0.275	−0.16	0.512	−0.239	0.324	0.085	0.729
SF36gh	19	0.15	0.539	0.118	0.629	0.054	0.827	−0.08	0.744	−0.008	0.974	−0.095	0.698	−0.019	0.937	−0.183	0.454	0.172	0.481
SF36hc	19	0.438	0.061	**0.504**	**0.028**	0.082	0.738	0.303	0.207	0.401	0.089	**0.572**	**0.011**	0.284	0.24	0.386	0.103	−0.174	0.476

Clinical scores	Cholin																		
	*n*	RFL		LFL		RTL		LTL		RPL		LPL		ROL		LOL		Cbl	
		*R*	*p*	*R*	*p*	*R*	*p*	*R*	*p*	*R*	*p*	*R*	*p*	*R*	*p*	*R*	*p*	*R*	*p*
B																			
L-HPT (s)	19	−0.312	0.193	−0.316	0.188	−0.479	0.038	−0.389	0.091	**−0.465**	**0.045**	−0.274	0.257	**−0.47**	**0.042**	−0.132	0.591	0.105	0.668
R-HPT (s)	19	−0.37	0.119	−0.255	0.293	−0.323	0.177	−0.377	0.112	−0.2	0.411	−0.225	0.355	0.364	0.125	−0.199	0.413	−0.089	0.718
TWT	19	−0.247	0.309	−0.363	0.126	−0.212	0.383	**−0.489**	**0.034**	−0.333	0.163	−0.312	0.193	**−0.554**	**0.014**	−0.225	0.355	−0.111	0.65
NEOS	18	−0.048	0.849	0.081	0.749	−0.244	0.33	−0.013	0.959	−0.086	0.734	−0.028	0.911	−0.067	0.791	0.126	0.618	0.236	0.347
SF36pf	19	0.055	0.823	−0.188	0.442	−0.134	0.583	−0.038	0.876	−0.07	0.776	0.079	0.747	0.014	0.955	0.142	0.562	−0.205	0.399
SF36rp	19	−0.015	0.95	−0.201	0.41	−0.014	0.954	−0.066	0.788	−0.08	0.744	−0.094	0.703	0.146	0.55	0.079	0.747	0.076	0.759
SF36re	19	−0.061	0.805	−0.301	0.211	−0.121	0.621	−0.164	0.503	−0.204	0.402	−0.121	0.621	0.048	0.845	0.083	0.736	−0.028	0.91
SF36e	19	0.124	0.614	0.033	0.894	0.12	0.624	0.026	0.917	0.147	0.549	−0.056	0.821	0.308	0.199	0.252	0.298	0.024	0.923
SF36wb	19	0.134	0.585	−0.013	0.957	−0.067	0.785	0.027	0.912	−0.157	0.521	0.02	0.934	0.179	0.464	0.239	0.325	0.137	0.575
SF36sf	19	0.009	0.971	−0.243	0.315	0.024	0.924	−0.053	0.828	0.037	0.88	−0.054	0.825	0.247	0.308	0.127	0.605	0.034	0.889
SF6p	19	0.105	0.667	0.151	0.537	0.278	0.249	0.008	0.974	0.118	0.632	0.051	0.837	0.095	0.698	0.024	0.921	0.4	0.09
SF36gh	19	0.158	0.518	0.022	0.928	0.01	0.969	0.006	0.98	−0.038	0.877	0.121	0.622	0.058	0.813	0.152	0.535	−0.138	0.574
SF36hc	19	−0.359	0.131	−0.28	0.246	−0.415	0.077	−0.377	0.111	−0.168	0.492	−0.105	0.67	−0.271	0.262	−0.087	0.724	−0.3	0.212

*p < 0.05, **p < 0.01, ***p < 0.001, ****p = 0.000.

Statistical significance is indicated by bold font. Reported *p*-values are uncorrected for multiple comparisons.

Anti-NMDA Encephalitis One-Year-Outcome-Score (NEOS-Score), Bodily Pain (SF-36-P), Cerebellum (CBL), General Health (SF-36-GH), Health Change (SF-36-HC), Left Frontal Lobe (LFL), Left Occipital Lobe (LOL), Left Parietal Lobe (LPL), Left Temporal Lobe (LTL), Left-Hand 9-Hole-Peg-Test (L-HPT), Mental Health (Well-being) (SF-36-WB), N-Acetyl-Aspartate (NAA), Physical Functioning (SF-36-PF), Right Frontal Lobe (RFL), Right Occipital Lobe (ROL), Right Parietal Lobe (RPL), Right Temporal Lobe (RTL), Right-Hand 9-Hole-Peg-Test (R-HPT), Role Limitations due to Emotional Problems (SF-36-RE), Role Limitations due to Physical Health (SF-36-RP), Social Functioning (SF-36-SF), Timed 25-Foot Walk Test (TWT), Vitality (Energy/Fatigue) (SF-36-E).

All reported *p*-values are uncorrected for multiple comparisons.

## Discussion

The study investigated variations in metabolite concentrations among individual brain lobes in patients after the acute phase of anti-NMDAR encephalitis. The most widespread differences were observed in Cho levels, with changes detected in four out of nine investigated regions. Cho plays an important role in cellular membrane turnover. Furthermore, the uptake of Cho serves as a crucial regulatory mechanism in the brain, influencing the synthesis of acetylcholine, a vital neurotransmitter involved in hyper- and depolarization within cells (20, 21). Elevated Cho concentrations are commonly interpreted as reflecting altered membrane turnover, cellular remodeling, or inflammatory tissue changes, a phenomenon that has been previously documented in other neuroinflammatory disorders, such as multiple sclerosis (22). The association between increased Cho concentrations in the left hemisphere and the timed 25-foot walk test performance suggests a potential relationship between regional metabolic alterations and motor impairment. This test is primarily used to examine patients with multiple sclerosis. However, a representative healthy control group was also examined in the description of the test, the results of which can be used for comparison with our patient collective. The median normal value was 3.7 s (2.8–5.2) (17). The median time taken by our patients was, on average, several seconds longer (on average 6.2 s; 4.04–9.6 s). The results of the nine hole peg tests are comparable to those of a healthy non-specific control group as documented in the literature (18). However, the observed correlation between left-hand 9-hole peg test performance and Cho concentrations in the right occipital lobe should be interpreted with caution. Despite the fact that the 9-hole peg test demands intact visuomotor integration, the functional relevance of this specific regional association remains uncertain. The elevated Cho concentrations observed in the patient cohort occurred in brain regions that have previously been reported to show reduced glucose metabolism on FDG-PET during the acute phase of anti-NMDAR encephalitis. Despite the heterogeneity of the studies and imaging modalities from which these findings originate, precluding direct correlation, they may imply the persistence of metabolic alterations after the acute phase, which could be indicative of ongoing remodeling processes (23, 24). In this context, our observation of regionally increased Cho concentrations may extend the currently limited MRS literature in anti-NMDAR encephalitis, in which Cho changes have been described only inconsistently and mostly in lesion-based or case-report settings (25).

MI as a marker for glia-cell especially astrocyte activation levels had altered levels in three lobes. Myo-Inositol is a well-established marker for glial cell activity, its increased concentrations in these areas may signify astrocytosis or microglial activation as components of the immunological response (26). As demonstrated in previous FDG-PET studies, occipital glucose hypometabolism has been identified as a hallmark of anti-NMDAR encephalitis. Moreover, the normalization of glucose metabolism has been shown to be concomitant with clinical improvement (24). In the present cohort, elevated myo-inositol concentrations were associated with a higher NEOS score, suggesting that persistent glial alterations may be related to a less favourable clinical course. As these findings were obtained using different imaging modalities in different patient cohorts, they should be regarded as complementary rather than directly comparable. The wider spectral linewidth observed in the left temporal lobe should be also interpreted with caution, as linewidth primarily reflects local field homogeneity and spectral quality rather than a direct biological marker; it may nonetheless indirectly relate to susceptibility effects near this brain region and represents a technical limitation to be considered when interpreting adjacent metabolite findings in this area. Our wbMRSI findings therefore expand previous single-voxel MRS observations by indicating that mI alterations may not be restricted to predefined frontal or temporal target regions but can also be detected across larger lobar brain regions (27). Rather than directly reflecting FDG-PET hypometabolism, increased mI may represent a complementary marker of post-inflammatory glial activation or tissue remodeling in regions that may appear structurally normal on conventional MRI without the need for radiation exposure of the patient. As tCr is widely regarded as a marker of cellular energy metabolism, the higher tCr concentrations observed in the left frontal and occipital regions may indicate persistent alterations in cerebral energy metabolism (28). A similar increase has been reported in the normal-appearing white matter of patients diagnosed with multiple sclerosis. Conversely, reduced tCr has been described in acute inflammatory lesions (29). This finding may be consistent with persistent glial alterations following anti-NMDAR encephalitis. As tCr is regarded as an indicator of cellular energy metabolism, elevated tCr concentrations may be indicative of altered metabolic activity as opposed to a particular biological process. Despite the pivotal role of astrocytes in sustaining brain homeostasis and responding to injury, the cellular mechanisms underlying the observed tCr increases remain undetermined based on the available data (11). This is of particular interest in the context of elevated tCr and persistent cognitive deficits. However, data on tCr alterations in anti-NMDAR encephalitis are scarce, and tCr has not yet been established as a consistent metabolic marker of disease activity or outcome in this condition. Therefore, these findings should be interpreted with caution and require confirmation in broader unaffiliated cohorts. The reduced NAA concentration observed in the area of the right temporal lobe may indicate regionally reduced neuronal integrity or neuronal dysfunction in patients following anti-NMDAR encephalitis. As a marker for the functional integrity and density of neurons, NAA shows region-specific changes. In addition to the cerebral cortex, the highest density of NMDA receptors is found in the hippocampal structures of the temporal lobe (30). The observed lateralization is consistent with previous reports of hemispheric metabolic asymmetries in anti-NMDAR encephalitis, although the underlying biological mechanisms remain uncertain (31, 32).

The hippocampus and medial temporal lobe are considered particularly susceptible to anti-NMDAR encephalitis because of their high density of NMDA receptors (30). Given the systemic nature of anti-NMDAR encephalitis, the occurrence of regional metabolic differences is to be expected. This finding lends further support to the hypothesis that sustained inflammation may result in a compromise of neuronal integrity even in the absence of overt structural abnormalities on conventional MRI (33).

The significantly higher scores in BDI II are consistent with clinical observations of an increased tendency towards depressive mood after anti-NMDAR encephalitis.

The correlation between BDI II results and physical limitations in CNS inflammation has already been described in the literature (34). In the case of the patients examined, however, there are no physical deficits for everyday living, which leads us to conclude that reactive depression is not a likely explanation. Reduced cognitive performance, illustrated here using the DemTect as an example, is also associated with persistent cognitive deficits after anti-NMDAR encephalitis as described above. However, no significant correlations between depressive symptoms and metabolite concentrations were observed. We also found no correlation between metabolite concentrations in individual brain lobes and SF-36 item results. Nevertheless, an average subjective assessment of general health was recorded in the patient group. Physical functioning was rated very positively, whilst emotional and social functioning, as well as the subjective status of mental health, were below average. This finding lends further support to the hypothesis that persistent depressive symptoms do not constitute a reactive phenomenon in relation to a residual physical impairment.

This study is limited by the lack of correction of the results for age and different time periods of measurement after encephalitis had passed. However, the age effect was minimized by matching patients and control subjects in terms of age and gender. Another limitation is that motor performance was only compared descriptively with published normative data, rather than with a healthy control group matched for age and sex. Furthermore, the available normative data were derived from individuals aged 25–29 years, whereas the patient cohort in the present study ranged in age from 18 to 64 years. Therefore, it is advisable to interpret these comparisons with care.

A further limitation is that the metabolite estimates were not further corrected for the relative proportions of gray and white matter. The analytical pipeline used was based on prior methodological work with the same MRSI processing and quantification approach including the exclusion of voxels with a CSF fraction >0.3 to minimize partial-volume effects (12).

The exploratory design, including multiple regional metabolite comparisons and clinical correlation analyses, should also be acknowledged, particularly because no correction for multiple comparisons was applied. Therefore, the observed associations between metabolite concentrations and clinical outcome measures should be interpreted as hypothesis-generating.

In view of the exploratory nature of the study and the number of statistical comparisons performed, this finding may represent an incidental association and requires confirmation in larger independent cohorts.

Furthermore, repeated comparisons of inflammation in the context of multiple sclerosis must also be viewed with caution, since the pathophysiological processes between autoimmune encephalitis and demyelinating disease differ and neuroinflammation is a general term.

It is also possible to regard a relatively small number of cases as limiting, although this must be considered in relation to the rarity of the disease.

The cognitive tests employed in our research align with extant findings concerning persistent cognitive deficits in anti-NMDAR encephalitis. Furthermore, we were able to demonstrate, that differences in brain metabolite concentrations are detectable even more than 1 year after the onset of acute inflammation and that the metabolite changes extend beyond the limbic system. This finding underscores the systemic nature of central nervous inflammation in the context of anti-NMDAR encephalitis. Further studies would be useful to analyze the metabolite concentrations in parallel with the clinic of patients during the course of the disease, in order to possibly derive prognostic parameters from the metabolite concentrations already in the acute inflammation phase.

## Data Availability

The original contributions presented in the study are included in the article/Supplementary material, further inquiries can be directed to the corresponding author.
