# Supplementary material for: Whole brain MR spectroscopy in patients with anti-NMDAR encephalitis
Source: Front Neurol. 2026 Sep 24;17:1892827. doi: 10.3389/fneur.2026.1892827 (PMC13645561; doi:10.3389/fneur.2026.1892827)
Supplement: Supplementary file 1 [file Table_1.docx]

| **Suppl. Table 1A** |  |  |  |  |  |  |  |  |  |  |  |  |  |  |  |  |  |  |  |
| --- | --- | --- | --- | --- | --- | --- | --- | --- | --- | --- | --- | --- | --- | --- | --- | --- | --- | --- | --- |
|  | **total Creatine** | | | | | | | | | | | | | | | | | | |
| **clinical scores** |  | **RFL** | | **LFL** | | **RTL** | | **LTL** | | **RPL** | | **LPL** | | **ROL** | | **LOL** | | **Cbl** | |
|  | ***n*** | ***R*** | ***p*** | ***R*** | ***p*** | ***R*** | ***p*** | ***R*** | ***p*** | ***R*** | ***p*** | ***R*** | ***p*** | ***R*** | ***p*** | ***R*** | ***p*** | ***R*** | ***p*** |
| **L-HPT (s)** | 19 | 0.018 | 0.943 | 0.088 | 0.721 | -0.114 | 0.642 | -0.104 | 0.673 | 0.144 | 0.557 | 0.242 | 0.318 | 0.019 | 0.937 | 0.123 | 0.616 | 0.284 | 0.238 |
|  |  |  |  |  |  |  |  |  |  |  |  |  |  |  |  |  |  |  |  |
| **R-HPT (s)** | 19 | 0.162 | 0.506 | 0.228 | 0.347 | 0.085 | 0.729 | 0.145 | 0.554 | 0.313 | 0.193 | 0.396 | 0.093 | 0.25 | 0.302 | 0.288 | 0.232 | 0.275 | 0.255 |
|  |  |  |  |  |  |  |  |  |  |  |  |  |  |  |  |  |  |  |  |
| **TWT** | 19 | 0.246 | 0.311 | 0.085 | 0.729 | 0.209 | 0.391 | 0.182 | 0.457 | 0.265 | 0.273 | 0.255 | 0.291 | 0.054 | 0.828 | 0.154 | 0.53 | 0.384 | 0.104 |
|  |  |  |  |  |  |  |  |  |  |  |  |  |  |  |  |  |  |  |  |
| **NEOS** | 18 | -0.137 | 0.589 | 0.081 | 0.749 | -0.212 | 0.398 | -0.201 | 0.423 | 0.161 | 0.522 | 0.1 | 0.693 | -0.016 | 0.948 | 0.045 | 0.86 | -0.155 | 0.538 |
|  |  |  |  |  |  |  |  |  |  |  |  |  |  |  |  |  |  |  |  |
| **SF36pf** | 19 | 0.122 | 0.618 | 0.148 | 0.547 | 0.108 | 0.659 | 0.07 | 0.776 | 0.207 | 0.394 | 0.259 | 0.285 | 0.048 | 0.846 | 0.22 | 0.365 | 0.252 | 0.298 |
|  |  |  |  |  |  |  |  |  |  |  |  |  |  |  |  |  |  |  |  |
| **SF36rp** | 19 | 0.096 | 0.697 | 0.076 | 0.756 | 0.229 | 0.345 | 0.287 | 0.234 | 0.162 | 0.509 | 0.011 | 0.963 | 0.109 | 0.657 | 0.227 | 0.351 | 0.423 | 0.071 |
|  |  |  |  |  |  |  |  |  |  |  |  |  |  |  |  |  |  |  |  |
| **SF36re** | 19 | 0.039 | 0.876 | -0.023 | 0.925 | 0.106 | 0.666 | 0.119 | 0.626 | 0.075 | 0.76 | -0.013 | 0.956 | -0.11 | 0.654 | 0.038 | 0.879 | 0.24 | 0.323 |
|  |  |  |  |  |  |  |  |  |  |  |  |  |  |  |  |  |  |  |  |
| **SF36e** | 19 | 0.132 | 0.591 | 0.133 | 0.588 | 0.162 | 0.508 | 0.399 | 0.091 | 0.027 | 0.911 | -0.066 | 0.787 | 0.113 | 0.645 | 0.268 | 0.268 | 0.188 | 0.44 |
|  |  |  |  |  |  |  |  |  |  |  |  |  |  |  |  |  |  |  |  |
| **SF36wb** | 19 | 0.119 | 0.628 | 0.132 | 0.59 | 0.089 | 0.717 | 0.138 | 0.572 | -0.063 | 0.799 | -0.019 | 0.937 | 0.176 | 0.471 | 0.222 | 0.361 | 0.422 | 0.072 |
|  |  |  |  |  |  |  |  |  |  |  |  |  |  |  |  |  |  |  |  |
| **SF36sf** | 19 | 0.058 | 0.814 | -0.027 | 0.912 | 0.195 | 0.425 | 0.288 | 0.232 | 0.048 | 0.845 | -0.062 | 0.8 | 0.015 | 0.95 | 0.081 | 0.74 | 0.24 | 0.323 |
|  |  |  |  |  |  |  |  |  |  |  |  |  |  |  |  |  |  |  |  |
| **SF6p** | 19 | 0.027 | 0.911 | 0.239 | 0.324 | 0.206 | 0.398 | 0.185 | 0.449 | 0.418 | 0.075 | 0.119 | 0.629 | 0.354 | 0.137 | 0.274 | 0.257 | **0.762** | **0.000** |
|  |  |  |  |  |  |  |  |  |  |  |  |  |  |  |  |  |  |  |  |
| **SF36gh** | 19 | 0.317 | 0.186 | 0.262 | 0.278 | 0.236 | 0.331 | 0.134 | 0.584 | 0.197 | 0.419 | 0.215 | 0.377 | 0.366 | 0.124 | 0.263 | 0.276 | 0.426 | 0.069 |
|  |  |  |  |  |  |  |  |  |  |  |  |  |  |  |  |  |  |  |  |
| **SF36hc** | 19 | -0.095 | 0.699 | 0.07 | 0.777 | -0.172 | 0.48 | -0.129 | 0.598 | 0.196 | 0.421 | 0.431 | 0.066 | 0.137 | 0.575 | 0.036 | 0.883 | -0.184 | 0.45 |
|  |  |  |  |  |  |  |  |  |  |  |  |  |  |  |  |  |  |  |  |
| **Suppl. Table 1B** |  |  |  |  |  |  |  |  |  |  |  |  |  |  |  |  |  |  |  |
|  | **Myo-Inositol** | | | | | | | | | | | | | | | | | | |
| **clinical scores** |  | **RFL** | | **LFL** | | **RTL** | | **LTL** | | **RPL** | | **LPL** | | **ROL** | | **LOL** | | **Cbl** | |
|  | ***n*** | ***R*** | ***p*** | ***R*** | ***p*** | ***R*** | ***p*** | ***R*** | ***p*** | ***R*** | ***p*** | ***R*** | ***p*** | ***R*** | ***p*** | ***R*** | ***p*** | ***R*** | ***p*** |
| **L-HPT (s)** | 19 | 0.1 | 0.684 | 0.088 | 0.721 | 0.24 | 0.322 | -0.046 | 0.853 | -0.026 | 0.915 | 0.114 | 0.642 | -0.067 | 0.786 | 0.291 | 0.226 | -0.012 | 0.96 |
|  |  |  |  |  |  |  |  |  |  |  |  |  |  |  |  |  |  |  |  |
| **R-HPT (s)** | 19 | -0.062 | 0.8 | 0.085 | 0.729 | 0.378 | 0.11 | -0.069 | 0.778 | 0.006 | 0.98 | 0.177 | 0.468 | -0.175 | 0.474 | 0.239 | 0.325 | -0.213 | 0.38 |
|  |  |  |  |  |  |  |  |  |  |  |  |  |  |  |  |  |  |  |  |
| **TWT** | 19 | -0.268 | 0.268 | -0.271 | 0.261 | 0.026 | 0.915 | -0.238 | 0.327 | -0.211 | 0.385 | -0.185 | 0.448 | -0.36 | 0.13 | 0.093 | 0.705 | -0.299 | 0.213 |
|  |  |  |  |  |  |  |  |  |  |  |  |  |  |  |  |  |  |  |  |
| **NEOS** | 18 | 0.314 | 0.204 | 0.204 | 0.417 | 0.164 | 0.516 | 0.38 | 0.119 | 0.362 | 0.14 | 0.312 | 0.207 | **0.51** | **0.031** | 0.166 | 0.51 | 0.436 | 0.071 |
|  |  |  |  |  |  |  |  |  |  |  |  |  |  |  |  |  |  |  |  |
| **SF36pf** | 19 | 0.095 | 0.698 | -0.021 | 0.93 | 0.133 | 0.588 | -0.044 | 0.858 | -0.176 | 0.472 | 0.033 | 0.894 | -0.169 | 0.489 | 0.094 | 0.701 | -0.107 | 0.662 |
|  |  |  |  |  |  |  |  |  |  |  |  |  |  |  |  |  |  |  |  |
| **SF36rp** | 19 | -0.059 | 0.81 | -0.143 | 0.558 | 0.143 | 0.558 | -0.037 | 0.88 | -0.157 | 0.522 | -0.102 | 0.677 | -0.241 | 0.32 | -0.098 | 0.688 | -0.156 | 0.524 |
|  |  |  |  |  |  |  |  |  |  |  |  |  |  |  |  |  |  |  |  |
| **SF36re** | 19 | -0.025 | 0.919 | -0.203 | 0.404 | 0.005 | 0.984 | -0.128 | 0.601 | -0.225 | 0.353 | -0.163 | 0.505 | -0.33 | 0.167 | -0.185 | 0.448 | -0.212 | 0.384 |
|  |  |  |  |  |  |  |  |  |  |  |  |  |  |  |  |  |  |  |  |
| **SF36e** | 19 | -0.141 | 0.566 | 0.004 | 0.986 | -0.047 | 0.849 | 0.08 | 0.743 | -0.095 | 0.7 | -0.019 | 0.94 | -0.167 | 0.494 | -0.035 | 0.886 | -0.249 | 0.303 |
|  |  |  |  |  |  |  |  |  |  |  |  |  |  |  |  |  |  |  |  |
| **SF36wb** | 19 | -0.004 | 0.989 | 0.022 | 0.929 | 0.025 | 0.92 | 0.124 | 0.612 | -0.207 | 0.395 | -0.041 | 0.866 | 0.045 | 0.855 | 0.151 | 0.538 | 0.024 | 0.923 |
|  |  |  |  |  |  |  |  |  |  |  |  |  |  |  |  |  |  |  |  |
| **SF36sf** | 19 | -0.148 | 0.547 | -0.215 | 0.376 | 0.043 | 0.863 | -0.129 | 0.597 | -0.316 | 0.188 | -0.241 | 0.321 | -0.395 | 0.095 | -0.253 | 0.295 | -0.311 | 0.194 |
|  |  |  |  |  |  |  |  |  |  |  |  |  |  |  |  |  |  |  |  |
| **SF6p** | 19 | 0.118 | 0.632 | 0.043 | 0.863 | **0.564** | **0.012** | 0.441 | 0.059 | 0.256 | 0.291 | 0.205 | 0.4 | 0.226 | 0.352 | 0.198 | 0.417 | 0.355 | 0.136 |
|  |  |  |  |  |  |  |  |  |  |  |  |  |  |  |  |  |  |  |  |
| **SF36gh** | 19 | -0.227 | 0.35 | -0.174 | 0.476 | -0.093 | 0.706 | 0.026 | 0.917 | -0.239 | 0.325 | -0.098 | 0.69 | 0.06 | 0.807 | 0.148 | 0.544 | 0.005 | 0.983 |
|  |  |  |  |  |  |  |  |  |  |  |  |  |  |  |  |  |  |  |  |
| **SF36hc** | 19 | -0.151 | 0.538 | -0.088 | 0.721 | 0.026 | 0.915 | -0.116 | 0.638 | -0.087 | 0.724 | 0.139 | 0.57 | -0.151 | 0.538 | 0.172 | 0.48 | -0.175 | 0.473 |
|  |  |  |  |  |  |  |  |  |  |  |  |  |  |  |  |  |  |  |  |

Suppl. Table 1 A-B: Presentation of the results of the nonparametric Spearman's correlation test of the average concentration for total Creatine (A) and Myo-Inositol (B)

in the individual brain regions with the results of the clinical tests performed. (p<0.05 *, p<0.01 **, p<0.001 ***, p=0.000 ****).

Statistical significance is indicated by bold font. Reported p values are uncorrected for multiple comparisons.

Anti-NMDA Encephalitis One-Year-Outcome-Score (NEOS-Score), Bodily Pain (SF-36-P), Cerebellum (CBL), General Health (SF-36-GH), Health Change (SF-36-HC), Left Frontal Lobe (LFL), Left Occipital Lobe (LOL), Left Parietal Lobe (LPL), Left Temporal Lobe (LTL), Left-Hand 9-Hole-Peg-Test (L-HPT), Mental Health (Well-being) (SF-36-WB), N-Acetyl-Aspartate (NAA), Physical Functioning (SF-36-PF), Right Frontal Lobe (RFL), Right Occipital Lobe (ROL), Right Parietal Lobe (RPL), Right Temporal Lobe (RTL), Right-Hand 9-Hole-Peg-Test (R-HPT), Role Limitations due to Emotional Problems (SF-36-RE), Role Limitations due to Physical Health (SF-36-RP), Social Functioning (SF-36-SF), Timed 25-Foot Walk Test (TWT), Vitality (Energy/Fatigue) (SF-36-E)
